# Exploring attitudes toward taxation of sugar-sweetened beverages in rural Michigan

**DOI:** 10.1186/s41043-021-00259-6

**Published:** 2021-08-03

**Authors:** Andrea E. Bombak, Taylor E. Colotti, Dolapo Raji, Natalie D. Riediger

**Affiliations:** 1 Department of Sociology, University of New Brunswick, Tilley Hall, Room 9, Fredericton, NB E3B 5A3 Canada; 2 School of Health Sciences, Community Health Division, Central Michigan University, 1280 E Campus Dr, Mt Pleasant, MI 48859 USA; 3 Department of Food and Human Nutritional Sciences, Faculty of Agricultural and Food Sciences, University of Manitoba, 407 Human Ecology Building, Winnipeg, MB R3T 2N2 Canada; 4 Department of Community Health Sciences, Rady Faculty of Health Sciences, University of Manitoba, Winnipeg, MB Canada

**Keywords:** Sugar-sweetened beverages, Tax, Attitudes, Obesity, Michigan

## Abstract

**Background:**

While policies to address “obesity” have existed for decades, they have commonly focused on behavioral interventions. More recently, the taxation of sugar-sweetened beverages is gaining traction globally. This study sought to explore individuals’ attitudes and beliefs about sugar-sweetened beverages being taxed in a rural Michigan setting.

**Methods:**

This qualitative study was conducted using critical policy analysis. Data were collected in 25 semi-structured, audio-recorded interviews with adult Michiganders. Following data collection, transcripts were coded into themes using NVivo software.

**Results:**

Four themes emerged in participants’ perspectives regarding sugar-sweetened beverages being taxed: *resistance*, *unfamiliarity, tax effects*, and *need for education.* While some participants were unfamiliar with sugar-sweetened beverage taxes, many viewed taxation as a “slippery slope” of government intervention, which invoked feelings of mistrust. In addition, participants predicted a sugar-sweetened beverage tax would be ineffective at reducing intake, particularly among regular consumers, who were frequently perceived as mostly low income and/or of higher weight.

**Conclusions:**

Further research is needed to explore perceptions of sugar-sweetened beverage taxes in different geographic areas in the USA to examine how perceptions vary. Policymakers should be aware of the potential implications of this health policy with respect to government trust and stigma towards lower income and higher-weight individuals.

## Introduction

“Obesity”^1^ [1] has been a topic of public health interest for decades, though its health implications are still under debate [2, 3]. While public nutrition education and physical activity campaigns have been the intervention of choice to date; more recently, fiscal policies have become a politically salient option [4], primarily the taxation of sugar-sweetened beverages (SSB^2^). The extent to which governments globally are considering the taxation of SSB as a policy measure to address “obesity” was invigorated in 2016 by the endorsement of the World Health Organization [5]. Despite adoption in some counties and its frequent endorsement by public health bodies, SSB taxation remains controversial [6], and the implementation of a SSB tax was recently repealed in Cook County, USA [7]. This study sought to explore individuals’ attitudes and beliefs about SSB being taxed in rural Michigan using a critical policy analysis approach.

### Public health agenda

Public health’s aim in introducing SSB taxes is to reduce the prevalence of “obesity”. The Centers of Disease Control and Prevention currently report that, presently, about 40% of adults in the USA are classified as “obese” [8]. Epidemiological studies link SSB consumption to weight gain and “obesity” in children and adults, although study findings are mixed [9]. Most added sugars in children’s and adolescent’s diets are from SSB [10]. As such, SSB have emerged as a major focus of policy interventions. While such taxes are often colloquially referred to as “soda taxes”, other SSB will generally be taxed including sport or energy drinks, sweetened tea, and beverages containing 50% or less fruit and vegetable juice. Unsweetened teas, coffee, beverages containing milk, infant formula, soy/rice or similar milk substitutes, carbonated or non-carbonated water that contains no sweeteners, non-alcoholic drink mixes, and soft drinks when mixed and sold in an alcoholic drink are *generally* excluded from the tax. There is already a sales tax on SSB in most states, applied at the point-of-sale; an excise tax would act as a flat tax to increase SSB shelf prices [11]. A SSB excise tax has been enacted in some countries, as well as numerous American counties. Berkeley, California was the first American county to introduce a SSB excise tax in 2014; neighboring San Francisco, Oakland, and Albany, California later voted in favor of the tax in 2016.

### Framing

A major contributor to controversies concerning SSB taxation is how SSB taxation is framed. Framing “assign[s] meaning to and interpret[s], relevant events and conditions in ways that are intended to mobilize potential adherents and constituents, to garner bystander support, and to demobilize antagonists” [[12] p198]. The type of framing steers institutions to certain policy responses while impeding others and is an important component of policy divergence and debate [13]. A SSB tax is framed as a policy response to “obesity”, which itself is commonly framed as a product of personal irresponsibility [14]. However, concerns over economic well-being, equity, free choice, and stigmatization are competing frames in the SSB taxation debate [6, 15].

A distributional burden exists regarding excise taxes, meaning any excise tax placed on SSB would be regressive. Taxes are considered regressive when they impose a greater burden on those of lower income as compared to those with higher income [16]. Low-income families spend a larger proportion of their income on shelter, food, and transportation, which means an excise tax on SSB would decrease their ability to afford the basic necessities of life. Indeed, the regressive nature of SSB taxes is frequently cited as a concern among the public [17]. In Australia, concerns over the regressive nature of the tax and fears it would have a deleterious effect on the poor led to a rejection of the tax [18].

The majority of literature on SSB taxation is from a public health perspective, where SSB taxes are frequently lauded (e.g., American Heart Association [19]; American Public Health Association [20]; American Medical Association [21]). “Big food” (i.e., the food industry) is repeatedly constructed as damaging to public health and the adversary of public health [22]. This can ignore how public health interventions themselves can exacerbate inequities and cause stigmatization and censure [23, 24]. Critical policy analysis asks, “Whose values have been validated?” [[25] p136] (i.e., what values are reflected and reinforced in the policy process). The following paper adds to the limited evidence and analysis concerning the viewpoints of other stakeholders, including the public, on the “problem” of SSB consumption and acceptable policy solutions to said “problem”.

### Policy target populations

A large body of research shows that the social construction of target populations plays a key role in the policy-making process (see for example Schneider and Ingram [26]. Of central concern to SSB taxation concerns is how persons labelled “obese” are constructed. “Obesity” is frequently constructed in scientific, popular, and media accounts as a failure of weak-willed individuals who are burdens to broader society, sufficient numbers of whom exist to constitute an “epidemic” [14, 27, 28]. This stigmatizing construction endures despite research presenting far more nuanced links between fatness and health [2, 3]. Counter-frames concerning “obesity” exist. Saguy and Riley [29] explored how antiobesity researchers and activists and fat acceptance researchers competed in framing contests to frame “obesity” as an epidemic brought about through risky behaviors or a form of natural diversity, respectively. Both sides sometimes marshalled the framing of “obesity” as a disease, to either promote treatment or reduce perceived personal culpability [29]. Subsequent research demonstrates frames that emphasize the social justice imperative of accepting diverse bodies or freedom of consumption and responsibility are reactionary frames that counter hegemonic frames of the unhealthiness and unattractiveness of fat bodies [30].

 During the early 2000s, the framing of “obesity” in the media was shifting somewhat from “individualizing” the “problem” to focusing on systemic issues, such as a toxic food environment. However, these frames did not wholly disrupt the focus on individual responsibility; the food environment may be threatening, but individuals are constructed as willingly “giving in” to these risks, which produce problems in need of “fixing” [31, 32]. Incorporating the food environment in accounts of “obesity” reinforces other common discursive frames of “problematic” bodies. Fatness is often coded as a lower class or racialized affliction [33–35]. The need to rectify “problematic” bodies (poor, racialized, and fat bodies) is frequently used as a justification to create environments that would, in fact, be beneficial for all [36]. Dietary interventions also frequently focus on those “othered” by class standing or racialization [37]. Excessive sugar consumption among the poor and the need to control is a perennial concern [38, 39]. One manifestation of such paternalism are “sin taxes” high enough to dodge [40].

### Taxation as a policy instrument

Sin taxes as a policy instrument have been applied to many products over time, including alcohol, tobacco, and gambling, though the phrase “sin taxes” originated in the 1970’s in response to taxation applied to tobacco. From a political perspective, sin taxes are easier taxes to implement as they tend to be more accepted by voters and often receive considerable advocacy support [41]. Sin taxes are informed by behavioral economic theory and are considered generally unobjectionable methods of nudging consumers away from certain behaviors. While the focus of evaluation of sin taxes tends to center on revenue impacts and effects on consumption, an important but often overlooked component is an examination of the politics of the policy-making process, particularly the views of general citizens. As Dagan [42] describes, taxes can be a reflection of social norms and meaning, but taxes also shape social norms and meanings. The very act of considering an item for taxation draws on a “normative taxpayer”. Every tax, including taxable income, deductions, rates, etc., “make assumptions and pass judgement as to what is ‘normal’ and what is not; they all de facto decide what is to be considered part of who we are (and therefore not to be infringed on by taxation) and what should be shared with others (and therefore relevant for tax purposes)” [[42], p2541].

Proponents of SSB taxation cite the reductions in SSB sales following the implementation of a SSB tax as demonstrating evidence for effectiveness [11, 43]. This reflects the increasing focus on generating “evidence-based policy”. While evidence-based policy is purported to minimize the politics of the policy process, many have critiqued the approach [44, 45]. Importantly, one’s epistemology influences the value placed on evidence-based policy. Evidence-based policy creates a hierarchy of evidence, which constructivists assert as detrimental to policymaking; personal experiences, public opinion, and history are regarded as less valuable than economic simulations and statistical models [44]. As Newman emphasizes, it is unlikely evidence will lead to a single clearly superior political end-goal. Rather, contextually relevant and nuanced evidence that takes into account specific social, economic, and political conditions can lead to a greater understanding of political acceptability in informing political decision-making [44, 46].

### Rural America and political context

Cultural and political context, including history, are an important component of critical policy analysis [47]. “No matter how well a [policy, systems, and environmental] intervention is designed and implemented from a technical perspective, people’s experience of and responses to the intervention will ultimately determine its effectiveness on health” [[48] p1355]. It is therefore essential to explore responses to a proposed intervention framed as a means of reducing consumption of an “obesogenic” substance.

Rural America is gaining traction as an area of considerable social and political salience. Studies of rural culture suggest uptake of values of self-reliance and independence [49]. The triumph of Donald Trump’s 2016 presidential campaign in Michigan and other rural regions generated news coverage and speculation that this was an indicator of festering rural resentment over perceived unjust taxation, immigration, employment, and distribution of resources, respect, and power [50–52]. Rural populations face barriers to adopting public health recommendations as these areas tend to be characterized by understaffed local health departments, hospital closures, high rates of poverty, wide dispersion of recreational and healthcare facilities, limited access to grocery stores, and questionable water quality [53, 54]. As such, the implementation of public health interventions in rural areas to address health inequalities are strongly encouraged.

To date, SSB taxes in the USA have only ever been implemented in “blue” states, or those with democratic leadership: Pennsylvania, California, and Washington [55]. Politically, Michigan is regarded as a “swing state” where the two major political parties have similar levels of support among voters. Proposals to implement an SSB tax in Michigan have, to date, failed [7]. The sociopolitical environment in Michigan is further informed by the Flint water crisis. Flint is an economically depressed city with a large African American population. In 2014, the population was exposed to lead-tainted water, following the cost-saving decision to obtain water from the Flint River. Fifteen individuals have been charged for their role in the public health crisis, and at least 12 fatalities have resulted from a Legionnaire’s Disease outbreak [56]. The Environmental Protection Agency sharply censured multiple levels of government in their handling of the crisis [57]. The Michigan Civil Rights Commission released a report documenting the systemic racism that underlies the crisis [58]. While the urban Flint crisis generated considerable media attention, Michigan’s rural population has long been affected by similarly controversial political decision-making [59, 60]. Public feelings concerning policy decisions, such as trust in public officials [61], must be considered in light of these tensions.

Specific to food access, approximately 14.2% of Michiganders experience food insecurity, or lack of access, at times, to sufficient, nutritionally adequate food for a healthy, active life for all members of a household [62], largely due to poverty [63]. While Michigan has a slightly lower cost of living compared to the rest of the USA, the poverty rate is about 2.5% higher than the national average [62]. In 2019, over 1.5 million people in Michigan received assistance from the Supplemental Nutrition Assistance Program [63], and over 200,000 families received assistance from the Michigan Women, Infants, and Children Program [64]. Due to the COVID-19 pandemic in 2020, the prevalence of food insecurity in the USA increased even further, particularly among racialized populations and households with children [65]. Given the tumultuous sociopolitical context, higher burden of food insecurity and “obesity”, and the regressive nature of SSB taxation, Michigan is a particularly pertinent site for exploring new and divisive policies.

The purpose of this study was to explore individuals’ attitudes and beliefs about SSB being taxed in rural Michigan using a critical policy analysis approach. Specifically, our research questions are:

1. How is a SSB tax perceived by rural Michiganders?
2. What beverages and populations do rural Michiganders perceive are targets of a SSB tax?
3. What do rural Michiganders believe  are acceptable uses of potential revenue from a SSB tax?

## Methods

### Design

This qualitative study was conducted using semi-structured interviews. Field notes were recorded to capture missing visual cues during interviews. This study was approved by the first author’s Institutional Review Board.

### Setting

The study was conducted in a Michigan city in a county defined as almost 47% rural by the 2010 United States of America Census [66]. A public university resides in the city. Residents of the area predominantly self-identify as White (88.2%), while 3.9% are American Indian and Alaska Native, 3.8% are Hispanic or Latino, 2.8% are Black or African American, and under 3% of the population identified as being two or more races. The poverty level of the area is approximately 23%, and the county has a 30% “obesity” prevalence [63].

### Recruitment and inclusion criteria

Participants were recruited through multiple efforts, including posters and social media. As interviews proceeded, snowball sampling occurred. To be considered for this study, the ability to speak and understand English and being at least 18 years of age were necessary. Participants were provided an honorarium to compensate for travel, parking, child care, and time. All participants provided their individual informed consent, including consent for audio recording.

### Data collection

Audio-recorded, semi-structured interviews were conducted by the lead author (AB) in her office between February and April, 2018. Participants also completed a demographic questionnaire with questions regarding gender, age, education, and SSB consumption habits. The interview guide included questions focusing on participants’ understandings and attitudes toward taxation of SSB, the beverages, and people perceived to be targeted by such a tax, and if implemented, their thoughts on what tax revenue should/would be used for. As part of this study, we specifically analyzed participant responses to the following interview guide questions:

1. How does price affect foods you buy? How does that make you feel? How do you cope?
2. What have you heard about the sugar-sweetened beverage tax?
  - What sources did you hear that from?
3. How do you think the tax will affect people?
  - What drinks do you think are the focus of the tax? Why?
  - What specific people will be affected?

4. How would you feel about the tax being introduced in Michigan?

5. Where do you think the money from the tax would go? Where would you want it to go?

Following interviews, field notes were immediately captured regarding the visual and verbal cues not captured on the recorder, reflexive notes on researcher bias, and notes on the interactional nature of the interview [67]. Interview lengths ranged from 15 to 53 min, with the mean being 30 min. Saturation was achieved prior to the conclusion of data collection; as there was a division among participants regarding those who perceived the taxation of SSB as possibly effective and those who perceived the taxation of SSB as ineffective, interviews proceeded after saturation to fully capture this variation in perspectives.

### Analysis

Data were analyzed thematically [68]. Meaning units from the transcripts were assigned to particular analytical groupings, “codes”. These codes were grouped into larger categories, and categories were collapsed into “themes”. The first author constructed a coding book by hand-coding the initial interviews. Hand-coding proceeded by annotating the interviews with a mix of deductive coding procedures: in vivo coding (using participants’ own language to construct codes) and construct coding (constructing codes based on underlying meanings) [69]. Deductive procedures also informed this process; some codes were drawn from relevant literature in food and body studies [68, 70]. Transcripts and the code book were uploaded into NVivo 11 software. Two authors were provided with the master code book and analyzed interviews as the interviews proceeded. The first three authors met regularly to discuss the data collection, analysis, and any possible code drift. These regular meetings allowed for an evolving research design, in which new codes could be incorporated and new themes could be probed for in future interviews [71–73].

Following initial coding, tables were constructed to identify patterns across participant characteristics, including age, gender, SSB drinker or non-drinker, etc., for every research and interview question. Summaries for every participant were constructed by the two analysts for each participant that described responses for every research and interview question.

## Results

Study participants (*n* = 25) were adults, men (24%) and women (76%), ranging in age from 18 to 60+ years, with a median age of 22 years. Approximately 88% of participants were Caucasian. Only 8% of participants reported never drinking SSB, 16% drinking SSB less than once per week, 36% drinking SSB one to three times per week, and 20% drinking SSB either four to six times per week or at least once per day. Due to the small sample size, demographic details (Table 1) are kept vague for the sake of confidentiality.

**Table 1:** Demographic characteristics of study participants

Age (years)	Sex (***n***)	Highest education level	Employment status	SSB consumption
18–60s	Female = 19	Completed secondary school = 2	Retired = 2	At least once per day = 5
	Male = 6	Some trade/technical school, college, university = 17	Semi-retired or working part-time = 11	4-6 Times per week = 5
		Completed trade/technical school or college diploma = 2	Working full-time in the labor force = 7	1-3 Times per week = 9
		Completed university degree = 2	Not working in the labor force = 5	Less than once per week = 4 Never = 2
		Some graduate education = 2		

Four major themes emerged in participants’ perspectives regarding SSB being taxed: *resistance*, *unfamiliarity, tax effects*, and *need for education/nutrition.* Themes are described below with relevant exemplar quotes, as well as summarized in Table 2.

**Table 2:** Description of themes and exemplar quotes.

Theme	Description	Quotes
Resistance	Expressions of negativity, opposition, ambivalence, or indifference regarding the tax on behalf of participants or their perception of others’ attitudes concerning the tax	“I think a lot of the opposition... to taxation of sugary beverages does kind of ... you're trying to control me. Don't tell me what to do.” – Linda “I think that it's a big deal that people are just like they let, let people do what they want. People don't want to be told what to do.” – Lisa “it might be a good thing, just because people they'll probably, maybe cut back on this certain drink, or…they gonna try to find those competitors, somebody that's not…on that same plan that everybody else is on…they might…go somewhere else…to find that sugar that they want from a pop or something like that. But I don't know. It's…wrong. Cause they tax us for everything, and life is already expensive. And to make something so little, that costs them very little to make…You wanna add, and an extra tax on our ass, that's stupid, and it's wrong.” -Trevor
Unfamiliarity	Lack of awareness of the proposed SSB tax or the debate concerning the tax potentially being introduced in the county; lack of familiarity with the details of the tax or taxation in general	**In response to interview question: “Have you heard anything about the sugar sweetened beverage tax?”** “No, not until you were doing this [the research study].” - Tracy “No…is there a proposal for tax on sugared beverages?” - Anne “not until like I saw the, the flyer... For this study. And I was like really surprised. I was like, ‘Oh, that's kind of interesting’.” - Shannon
Tax effects	Statements regarding the perceived likely effectiveness of SSB taxation, comparison to other policies in terms of effectiveness, or statements regarding who the tax would most likely affect	“I have mixed feelings…I think to make the world healthier it's a good idea cause it probably deters some people from buying it. But the same time, I know, for example, I have some friends, my mom, you could almost think they're like Coke-aholics…Where they need a can a day or my one friend probably needs three McDonald's drinks a day or else he'll get moody…So even then, I don't know if the tax will play that much role, unless it's a big tax, it'll, they'll have to pay the extra couple cents or a dollar.” – Michael “the government's taxing everything anyways, just like the cigarettes… they raised the price on them, raise the price on them, tax them and tax them, and is how many people quit because of the price, and it's probably not that many.” - Rita “probably heavy people [would be most affected by a SSB tax]”. - Melinda
Need for education	Desire for tax revenues to be used for health/nutrition education programs or school-based health/nutrition programs; general desire for more health/nutrition education programs; or expression of belief in such programs’ effectiveness	“But I like the idea of…program- like implementing like, each state get like, a certain amount, and like distribute that to…do education about…drinks and…everything. More than I got in health class about it just affecting your arteries.” – Elizabeth “For just maybe on like what I would like it to go to is like education on just nutrition in general…I think I would like to be more education on nutrition…as a child and as a student…I think that would be really cool, but. – Melissa “maybe something regarding, like…nutrition or health program or something like that. Maybe making more… opportunities available for people to access better nutrition or health…but also…the way the government is, you really have no idea where a lot of that money goes. So, I would hope that it would go towards nutrition and health programs for…even like schools ... Michelle Obama, she was really pushing for the whole…nutrition, so maybe programs of that nature. Or something related to health.” - Julia

### Resistance

Most participants expressed negativity, ambivalence, or indifference regarding the tax. Some participants referenced a belief that putting a tax on SSB is a “slippery slope” that implies the government is trying to control their decisions:

I don't think that'd be a good idea…where do you draw the line? I feel like… how does one person rule what…like, okay, well this isn't healthy, so I don't think you should have this…you know what I mean? I feel like that's a slippery slope (Brandon).

There is also resistance toward the government implementing a tax because people cannot trust how the revenue will be used:

We would see no result of it, just like everything else. The money’s supposed to go somewhere, and it never does (Lorraine).

For some participants, the SSB tax was another means by which the government would seek to curtail individual autonomy. Such means were often seen as being clouded in obfuscation or deception and were rarely seen to promote stated, agreed upon, or relevant priorities. Possibly due to resistance to government interference in personal decision-making for ambiguous aims, most participants did not appear supportive of government regulation. One participant tied this to a local social and political consciousness. Melinda believed “liberals” would support the tax as “they’re always fighting a cause” and believed that the tax would have less local support:

think that's only in New York. (laughs) No, I don't think we're in the areas…You know, where they ... we do that kind of stuff. We're middle America. We ... I think we kinda leave each other alone and we don't get into everybody's business, you know?

Although the majority of participants resisted a tax on SSB, a few participants, who drink pop regularly, thought a SSB tax would be a good idea to encourage healthier behaviors:

It'd probably be a good idea to keep people in line, healthier (Lisa).

Some participants also thought that a tax on SSB would make them consciously think about purchasing pop beforehand, and may even divert them from purchasing it all together:

It would make me say ‘Well, do I really want that pop? Do I really, you know, need that?’ Maybe it's a good thing, maybe…more people would feel that way and they would consume less of it (Amber).

### Unfamiliarity

In addition to government distrust, younger participants were sometimes unfamiliar with tax procedures, and many had never heard of SSB taxation:

I have no idea. I should probably … I don't pay attention to … I need to pay more attention to the news in general and just like the government, but I don't (Melissa)

The sugar-sweetened beverage tax? Nn-nnh (negative), actually I haven't (Katie).

There was also some confusion among participants of all ages as to how tax revenue is used by the government.

I don't know. Maybe, well wouldn't the money go back to … the sugar sweetened beverages people? (Lisa).

In general, participants had little awareness of intended or proposed SSB regulations, suggesting perhaps that this topic has not penetrated the public consciousness in this region. Some participants were also unsure how taxation operated. This might influence the overall effectiveness of the tax.

### Tax effects

Many participants felt an SSB tax would generate opposition among those who consume SSB, but that the tax would not necessarily alter behaviors:

I think it would cause a little bit of outrage just for the people who get pop on a regular basis. They're like, ‘Oh, my gosh, like why all of a sudden do I have to pay extra for this?’ But…I think if somebody really wants it, the tax isn't really gonna matter too much to them (Shannon).

Participants sometimes referenced taxes on cigarettes in expressing their doubt over the effectiveness of a prospective tax:

I don't think it'll impact it that tremendously... People in New York, they still buy cigarettes. I think it's like $15 a pack or something to buy cigarettes there. They're still smokin' (Denise)

Many participants think that lower-income individuals would be most affected by a SSB tax. In response to the question, “who is going to be most affected by the tax?” Linda succinctly stated, “poor people”. Anne similarly felt those of a lower-income would be most affected by the tax, although she qualified her answer by drawing on personal responsibility framing around food choices:

The ones who, well, probably shouldn't be drinking them anyway because if you have limited income then you need to use your money to make the healthiest choices you can but that should be for everybody that. But when you are buying pop at the expense of food with nutritional value... If you're buying both and providing both to your family, that's one thing but if you're buying one and now you can't afford the other one, that's a problem for me.

And so, but we know that's happening so raising the tax is only gonna hurt the poor people (Anne).

Aside from taxes affecting lower-income individuals more than those of a higher income, many participants think lower-income individuals would be affected most by a SSB tax because they believed SSB tended to be the cheapest option for persons of a lower income to drink:

I feel like lower income families would definitely oppose it because you're taking the cheapest, easiest accessible like option for them and then putting a tax on it. So, I almost feel like it would be negative for them (Tiffany).

Some participants, like Katie, further assume that SSB imbibers likely are of higher weights and therefore that higher-weight individuals be most affected by a tax:

Or like more obese people just because they, like, like pop better than water, which I don't get how, but still. I just feel like that's kind of like the stereotypical thing to say, like I said, but I feel like that's the image that media puts out, but that's what I'm thinking. You know people who are bigger, people who don't have as much money and they kind of want that extra treat. You know what I mean, like, they get 'em the pop.

Some participants did not differentiate among demographic groups and thought all who enjoy drinking SSB would be affected. Makayla emphasizes that the tax will not discriminate along any other social axis apart from personal preference:

anyone really who just loves pop, or loves all that sweet stuff, doesn't really matter if you're, race or class, or anything.

Makayla’s quote highlights a further pattern in the data—participants focused frequently on pop and believed pop would be the focus of governmental reforms, given its perceived unhealthfulness and popularity. For example, in response to a question regarding what beverages would be the target of the tax, Julia replied as follows:

probably pop? Because I think that…there's just kind of an idea that juice isn't as unhealthy. Which, I mean, could be true but there's definitely still a lot of sugar in things like orange juice and apple juice and what are people consider, like, typically healthier alternatives...I think they still definitely have sugar in them, but I think that people would ideally focus on pop because that's kind of what you think of automatically (Julia).

### The need for education

While participants tended to oppose a tax**,** if a SSB tax were to be implemented, many participants think revenue generated from a tax should be put toward health or nutrition education programs or health/nutrition school-based programs. Rita explains how children should be the priority population regarding these programs:

I think it would be nice to see it go to education type… maybe like for the young children, and that way they're learning about it, and just…like nutrition programs.

Participants felt children should be exposed to these programs at a very young age to deter them from ever drinking SSB in the first place.

They should be more concerned and telling, and teaching kids better habits when they're younger (Andrew).

However, reflecting the general skepticism regarding governance, participants were doubtful that the revenue would be used to address what they considered to be appropriate programs:

in my mind if you're gonna put a tax on something like that, let's do something good with that money…Educate people about nutrition. Educate people about why this is, you know important. Or… in the schools and maybe set up programs for the kids that… would be where it would be most used and, and beneficial. But, it doesn't tend to go that way.... Who knows where they'd put it (laughter)…let's do something good with this. But…our government doesn't always focus that way (Amber).

## Discussion

Our results suggest that public attitudes in Michigan towards SSB taxation may not be aligned with those of public health agendas. This misalignment may be partially attributed to perceived lack of effectiveness of such a policy, an over-reach on behalf of the government, or the edge of a dangerous slippery slope of government interference. Participants generally felt that pop or soda were the main targets of an SSB tax and that individuals with lower incomes, as well as people of a higher weight would be most affected. Though participants were generally opposed to an SSB tax, if such a policy were implemented, nutrition education, particularly directed at children, was considered an acceptable use of tax revenue.

Our findings of unfamiliarity and resistance toward an SSB tax are generally consistent with findings from other studies of acceptability. In a quantitative study conducted in Switzerland, taxation generated higher resistance levels among participants than less intrusive methods aimed at reducing population sugar intake, like front-of-package labeling [74]. In addition, a qualitative study, including Mexican adolescents who also reported being frequent consumers of SSB, found participants were generally unaware of the recent implementation of an SSB tax in their country. Furthermore, adolescent participants reported being doubtful of its effectiveness [75], similar to our findings (although see also Krukowski et al. [76]). Public health advocates may overestimate how much people care or notice taxes, particularly those who are frequent consumers. The lack of perception of SSB tax may be explained by public choice theory and the concept of fiscal illusion, or the failure to fully perceive the complete cost to the taxpayer [77].

Relevantly, research confirms that lower socioeconomic populations, across many countries, are higher consumers of SSB [78, 79]. A French study of acceptability of their SSB tax found that individuals with lower levels of education were significantly less likely to support an SSB tax and more likely to agree that a tax would be unfair [80]. Critically, however, SSB are consumed across socioeconomic strata; sugar-sweetened coffee intake is highest in American regions with higher White population and higher socioeconomic status [78]. The class and race implications of the framing and policy formulation of the “pop/soda tax” are therefore essential.

Participants’ responses revealed the ways in which public policy around SSB and taxation has constructed the “problem” of “obesity”. Participants identified high SSB consumers, those living on lower incomes, and higher-weight individuals as particularly likely to be affected by SSB taxation. Similar to findings reported above in which “pop” or “soda” are viewed as particularly dangerous to health, SSB coded as White and middle-class, were perceived as decadent but did not carry the same widespread risk connotations [81]. Crucially, participants also stated that they judge higher-weight individuals more for consuming SSB, and they associate SSB (particularly pop) with “obesity” and weight gain [81]. Thus, pop is discursively linked to body size, class, and a likely object of government intervention. As target populations, higher-weight and lower-income individuals have been constructed negatively and as politically weak. Such a construction helps to justify punishing or burdensome policies [82], such as a regressive excise tax.

The linkage between “obesity” and class may also be refracted through a lens of ignorance, such that fatness, the working classes, character deficits, and ignorance are conflated [83, 84]. Rural spaces also carry the presumption of non-cosmopolitan, unsophisticated, “obesogenic” backward, lower-class foodscapes [85, 86]. For example, in a study on the perceptions of local health threats in a rural context, a higher-than-average income sample, with some exceptions, linked moral and intellectual deficiencies to what they perceived as poor diets and insufficient exercise [84]. Intriguingly, with respect to rural adults, National Health and Nutrition Examination Survey (NHANES) data from 1999 to 2006 indicate rural adults consume more SSB than urban adults; however, light and heavy SSB consumption is not associated with “obesity”, and moderate consumption is associated with lower risk of “obesity” [87]. The focus on disseminating information as key to health outcomes [88] persisted in participants’ recommended use of revenue—nutrition education. However, some resistance to the belief that “Middle America” was in need of outsiders’ intervention was expressed by at least one participant and others were concerned that the tax represented the edge of a “slippery slope” of more interventions imposed by a government that was often distrusted.

While the purported goal of an SSB tax is to address “obesity”, a Swiss survey also found that people with increased health risks, higher-weight individuals, and those consuming higher amounts of SSB, are less accepting of a tax [74]. Similarly, a French study demonstrated that higher consumers of SSB were less likely to agree that an SSB tax would improve population health [80]. A rare qualitative study in the area found Michigan adolescents felt social censure and taxation would likely reduce their consumption, which they perceived as a positive, unless they frequently drank SSB [76]. However, rural stakeholders in North Carolina perceived the public as being disinclined toward “obesity”-prevention strategies deploying taxes, government mandates, or incentives; discouraging SSB consumption was rated particularly unwinnable [89]. Policymakers should be aware of lower levels of policy acceptability among populations targeted as this may forecast lower policy effectiveness. It may also be an indicator of resistance toward stigma that may be perpetuated by this policy, as we have previously reported  [81].

SSB taxes are frequently compared with tobacco taxes as both effective at reducing smoking as well as generating government revenue [90]. However, almost all tobacco revenue goes into a state’s general fund, with significant state variation in the funding provided to support smoking cessation programs [91]. Previous experience with tobacco taxation suggests participants’ skepticism of revenue being used for appropriate programs is warranted. Indeed, the lack of support for cessation while increasing tobacco taxes has been noted as a source of stigma for New Zealanders who smoke [92].

Results of the present study indicate that revenue use is an important issue. Research has also demonstrated the importance of acceptable revenue use in garnering political and public support for an SSB tax [80]. It has been reported that the reason SSB taxation passed in Philadelphia, PA, USA is that revenue use was earmarked for pre-kindergarten spots [93]. As such, public health advocates have increasingly turned their attention to identifying acceptable revenue uses, particularly uses that may enhance health equity. While suggested uses for tax revenue may be effective in improving health equity as well as acceptable, such as publicly funded pre-kindergarten [94], safe drinking water [95], and school lunch programs [96], the *inequity* in the revenue generation remains. Furthermore, attention must be paid to the inequity in negotiating power of marginalized populations in maneuvering this political quid pro quo, as well as the ethics of utilizing human rights, such as drinking water [95], to garner support for a controversial, regressive [97], and potentially stigmatizing public policy.

A common justification for implementing a tax on SSB is to use the revenue for the “common good”: children’s education, health promotion, etc.. Such recommendations may have to be interpreted with caution. Dietary behavior change interventions have produced mixed results [98]. Indeed, reporting issues may limit drawing conclusions of nutrition/lifestyle interventions [99, 100]. Educational interventions that focus on SSB have produced at best short-term, mixed results [101]. Findings suggest that individuals substitute other products in lieu of the targeted SSB [101]. Furthermore, such interventions focus on SSB and not “sugars” in general, which will always limit their utility [101]. Additionally, school food, weight, and body education strategies can be unintentionally stigmatizing and affect adolescents’ embodied well-being [102, 103]. Careful consideration of such programs, including a weight-neutral approach that does not ostracize or label some children’s bodies as “problems” is essential [104].

Participants in the present study perceived pop or soda as being the main targets of SSB taxes, despite a broader range of beverages included in most SSB taxes. This perception appears to align with both researcher and distributor perspectives of where tax pass-through should occur. “Pass-through” refers to the change in price following the implementation of an SSB tax. In an analysis of tax pass-through at the Philadelphia International Airport, which straddles the city boarder and hence the application of a SSB tax, only Coca-Cola and Pepsi-Cola beverages were selected to determine any differences in tax pass-through [105]. With respect to distributors, when a greater variety of SSB were selected to evaluate tax pass through in Berkeley, regular soda and energy drinks were observed to have the greatest tax pass-through at 1.09 cents/oz as compared with only 0.41 cents/oz for other taxed beverages that were fruit, vegetable, or tea flavored [106]. This may be reflective of an underlying belief of which drinks are tax targets and the social perceptions of those beverages, rather than the penny-per-ounce tax itself.

In conclusion, rural Michiganders perceived a tax on SSB with resistance, unfamiliarity, and ineffectiveness. Additional themes among participants included the SSB tax targeting pop, the tax affecting those of higher weights and lower incomes, and tax revenue being used for education. Further research is needed to explore perceptions on SSB taxes in different populations and geographic areas around the USA. Of particular importance would be those of lower income, those who drink SSB, and those who are of higher weight who are the most likely to be affected by the tax or are being framed as the “targets” of taxation.

## Data Availability

The datasets generated and/or analyzed during the current study are not publicly available due to ethical reasons, but are available from the corresponding author on reasonable request and corresponding institutional ethics board approval.

## Abbreviations

SSB: Sugar-sweetened beverage
NHANES: National health and nutrition examination survey

## Footnotes

1 Throughout this manuscript, obesity is presented in quotes in recognition of the political and contested nature of this category. Fat is used in a non-pejorative sense in line with its usage by fat acceptance advocates as a neutral description of human diversity (Meadows and Daníelsdóttir, [1]).

2 Sugar-sweetened beverages

## References

[1] Meadows A, Daníelsdóttir S (2016). What’s in a word? On weight stigma and terminology. Front Psychol..

[2] Bacon L, Aphramor L (2011). Weight science: evaluating the evidence for a paradigm shift. J Nutr.

[3] Campos P, Rich E, Monaghan LF, Aphramor L (2011). Does fat kill? A critique of the epidemiological evidence. Debating obesity.

[4] Tamir O, Cohen-Yogev T, Furman-Assaf S, Endevelt R (2018). Taxation of sugar sweetened beverages and unhealthy foods: a qualitative study of key opinion leaders’ views. Isr J Health Policy Res.

[5] Thow AM, Downs SM, Mayes C, Trevena H, Waqanivalu T, Cawley J (2018). Fiscal policy to improve diets and prevent noncommunicable diseases: from recommendations to action. Bull World Health Organ..

[6] Bogart WA (2013). Law as a tool in “the war on obesity”: useful interventions, maybe, but, first, what’s the problem?. J Law Med Ethics..

[7] VVB. Chicago’s soda tax repealed: A big victory for makers of sweet drinks. In: The Economist. 2017. https://www.economist.com/democracy-in-america/2017/10/13/chicagos-soda-tax-is-repealed. Accessed 21 July 2021.

[8] Centers for Disease Control and Prevention. Adult obesity facts. 2018 August 13. Atlanta, GA Available from: https://www.cdc.gov/obesity/data/adult.html. Accessed 14 Feb 2019.

[9] Malik VS, Schulze MB, Hu FB (2006). Intake of sugar-sweetened beverages and weight gain: a systematic review. Am J Clin Nutr.

[10] Reedy J, Krebs-Smith SM (2010). Dietary sources of energy, solid fats, and added sugars among children and adolescents in the United States. J Am Diet Assoc..

[11] Falbe J, Thompson HR, Becker CM, Rojas N, McCulloch CE, Madsen KA (2016). Impact of the Berkeley excise tax on sugar-sweetened beverage consumption. Am J Public Health..

[12] Snow DA, Benford RD (1988). Ideology, frame resonance, and participant mobilization. Int Soc Mov Res..

[13] Hawkins B, Holden C (2013). Framing the alcohol policy debate: industry actors and the regulation of the UK beverage alcohol market. Crit Policy Stud.

[14] Puhl RM, Heuer CA (2010). Obesity stigma: important considerations for public health. Am J Public Health..

[15] Riediger ND, Bombak AE, Mudryj A, Bensley J, Ankomah S (2018). A systematic search and qualitative review of reporting bias of lifestyle interventions in randomized controlled trials of diabetes prevention and management. Nutr J.

[16] Poterba JM. Lifetime incidence and the distributional burden of excise taxes: Working Paper Series No. 2833. In National Bureau of Economic Research. 1989. https://www.nber.org/system/files/working_papers/w2833/w2833.pdf. Accessed 21 July 2021.

[17] Amadeo, K. Regressive tax with examples, the balance. New York, NY; 2019. Available from: https://www.thebalance.com/regressive-tax-definition-history-effective-rate-4155620. Accessed 19 Mar 2019.

[18] Di Natale, R., L Singh, P. Georgiou, K. Kitching, J. Paterson, A. Stoker and T. Storer. Select committee into the obesity epidemic in Australia final report. Parliament of Australia. 2018 December 5. Available from: https://www.aph.gov.au/Parliamentary_Business/Committees/Senate/Obesity_epidemic_in_Australia/Obesity/Final_Report

[19] American Heart Association. Reducing sugar-sweetened beverage consumption a focus on sugar-sweetened beverage taxes. Washington, DC; 2016 June. Available from: https://www.heart.org/-/media/files/about-us/policy-research/prevention-nutrition/sugar-sweetened-beverage-taxation-ucm_490766.pdf?la=en&hash=78FF27BF18A0A7967526C4052FDA3DC267AA64DD. Accessed Jan 2020

[20] American Public Health Association. Taxes on sugar sweetened beverages. Washington, DC; 2012 Oct. Available from: https://www.apha.org/policies-and-advocacy/public-health-policy-statements/policy-database/2014/07/23/13/59/taxes-on-sugar-sweetened-beverages. Accessed 11 Jan 2020

[21] American Medical Association. AMA adopts policy to reduce consumption of sugar-sweetened beverages. Chicago; 2017 June. Available from: https://www.ama-assn.org/press-center/press-releases/ama-adopts-policy-reduce-consumption-sugar-sweetened-beverages. Accessed 11 Jan 2020

[22] Nestle M (2013). Food politics: How the food industry influences nutrition and health.

[23] Phelan JC, Link BG, Tehranifar P (2010). Social conditions as fundamental causes of health inequalities: theory, evidence, and policy implications. J Health Soc Behav..

[24] Langford R, Panter-Brick C (2013). A health equity critique of social marketing: where interventions have impact but insufficient reach. Social Sci Med..

[25] Prunty JJ (1985). Signposts for a critical educational policy analysis. Aust J Educ..

[26] Schneider AL, Ingram HM, Ingram HM. Deserving and entitled: social constructions and public policy. Ithaca: State University of New York Press; 2005.

[27] Gard M, Wright J. The obesity epidemic: science, morality and ideology. London: Routledge; 2005. 10.4324/9780203619308.

[28] Halse C. Bio-citizenship: virtue discourses and the birth of the bio-citizen. In Wright J Harwood, editors. Biopolitics and the 'Obesity Epidemic'. New York, NY. Routledge; 2012.p. 45-59

[29] Saguy AC, Riley KW (2005). Weighing both sides: morality, mortality, and framing contests over obesity. J Health Politics Policy Law.

[30] Kwan S, Graves J. Framing fat: competing constructions in contemporary culture. New Brunswick: Rutgers University Press; 2013.

[31] Lawrence RG (2004). Framing obesity: the evolution of news discourse on a public health issue. Harvard Int J Press/Politics..

[32] Cain P, Donaghue N, Ditchburn G (2017). Concerns, culprits, counsel, and conflict: a thematic analysis of “obesity” and fat discourse in digital news media. Fat Stud.

[33] Raisborough J. Fat bodies, health and the media. London: Palgrave Macmillian UK; 2016 May 24. 10.1057/978-1-137-28887-5.

[34] LeBesco K. Revolting bodies?: the struggle to redefine fat identity. Amherst: Univ of Massachusetts Press; 2004.

[35] Meeuf R (2016). Class, corpulence, and neoliberal citizenship: Melissa McCarthy on Saturday night live. Celebr Stud..

[36] Kirkland A (2011). The environmental account of obesity: a case for feminist skepticism. Signs..

[37] Guthman J (2011). Weighing in: obesity, food justice, and the limits of capitalism.

[38] Mulderrig J (2019). The language of ‘nudge’ in health policy: pre-empting working class obesity through ‘biopedagogy’. Crit Policy Stud..

[39] Mechling EW, Mechling J (1983). Sweet talk: the moral rhetoric against sugar. Commun Stud..

[40] Caplan B (2001). Rational ignorance versus rational irrationality. Kyklos..

[41] Johnson CM, Meier KJ (1990). The wages of sin: taxing America’s legal vices. West Polit Q..

[42] Dagan T. The currency of taxation. Fordham L Rev. 2015;84:2537.

[43] Colchero MA, Guerrero-López CM, Molina M, Rivera JA. Beverages sales in Mexico before and after implementation of a sugar sweetened beverage tax. PloS One. 2016;11(9):e0163463. 10.1371/journal.pone.0163463. 10.1371/journal.pone.0163463 PMC5036812 27668875

[44] Newman J (2017). Deconstructing the debate over evidence-based policy. Crit Policy Stud.

[45] Monaghan M (2010). The complexity of evidence: reflections on research utilisation in a heavily politicised policy area. Soc Policy and Soc..

[46] Mccoy D, Chigudu S, Tillmann T (2017). Framing the tax and health nexus: a neglected aspect of public health concern. Heal Econ Policy Law..

[47] Apple MW (2019). On doing critical policy analysis. Educ Policy..

[48] Kumanyika SK (2019). A framework for increasing equity impact in obesity prevention. Am J Public Health..

[49] Gessert C, Waring S, Bailey-Davis L, Conway P, Roberts M, VanWormer J (2015). Rural definition of health: a systematic literature review. BMC Public Health..

[50] Cramer KJ. The politics of resentment: rural consciousness in Wisconsin and the rise of Scott Walker. Chicago: University of Chicago Press; 2016. 10.7208/chicago/9780226349251.001.0001.

[51] Morin, R. Behind Trump’s win in rural white America: women joined men in backing him. Pew Research Center. 2016 Nov 17. Available from: http://www.pewresearch.org/fact-tank/2016/11/17/behind-trumps-win-in-rural-white-america-women-joined-men-in-backing-him/. Accessed 16 May 2017.

[52] Leonard, R. Why rural America voted for Trump. The New York Times. [Internet] 2017 January 5. [cited 2017 May 16] Available from: https://www.nytimes.com/2017/01/05/opinion/why-rural-america-voted-for-trump.html

[53] Harris JK, Beatty K, Leider JP, Knudson A, Anderson BL, Meit M (2016). The double disparity facing rural local health departments. Annu Rev Publ Health..

[54] Hess JM, Lilo EA, Cruz TH, Davis SM (2019). Perceptions of water and sugar-sweetened beverage consumption habits among teens, parents and teachers in the rural south-western USA. Public Health Nutr..

[55] Backholer K, Vandevijvere S, Blake M, Tseng M (2018). Sugar-sweetened beverage taxes in 2018: a year of reflections and consolidation. Public Health Nutr..

[56] Ruble, K., A. Ellis, J. Carah and S. Childress. Five years in, the Flint water crisis continues its deadly toll. PBS Frontline [Internet]. 2019 Apr 25 [cited 2019 Oct 12]. Available from: https://www.pbs.org/wgbh/frontline/article/flint-water-crisis-legionnaires-disease-deaths/

[57] United States Environmental Protection Agency. Management weaknesses delayed response to Flint water crisis [Internet]. 2018 Jul 19 [cited 2020 Jan 13]. Available from: https://www.epa.gov/sites/production/files/2018-07/documents/_epaoig_20180719-18-p-0221.pdf

[58] Michigan Civil Rights Commission. The Flint water crisis: systemic racism through the lens of Flint. 2017 Feb 17. Available from: https://www.michigan.gov/documents/mdcr/VFlintCrisisRep-F-Edited3-13-17_554317_7.pdf. Accessed 12 Oct 2019.

[59] Clark K (2016). The value of water: the Flint water crisis as a devaluation of natural resources, not a matter of racial justice. Environ Justice.

[60] Miller DS, Wesley N (2016). Toxic disasters, biopolitics, and corrosive communities: guiding principles in the quest for healing in Flint, Michigan. Environ Justice.

[61] Cuthbertson CA, Newkirk C, Ilardo J, Loveridge S, Skidmore M (2016). Angry, scared, and unsure: mental health consequences of contaminated water in Flint, Michigan. J Urban Health.

[62] United States Department of Agriculture. ERS - Food Access Research Atlas. 2019 [updated 2019 Oct 31]. Available from: https://www.ers.usda.gov/data-products/food-access-research-atlas/. Accessed 11 Jan 2020.

[63] United States Department of Agriculture. SNAP community characteristics – Michigan. 2019 [updated 2019 Aug 8]. Available from: https://www.fns.usda.gov/ops/snap-community-characteristics-michigan. Accessed 11 Jan 2020.

[64] Michigan Department of Health and Human Services. The WIC program. 2019 Feb 14. Available from: https://www.michigan.gov/mdhhs/0,5885,7-339-71547_4910_6329%2D%2D-,00.html. Accessed 20 Jan 2020.

[65] Schanzenbach D, Pitts A. How much has food insecurity risen? Evidence from the census household pulse survey. Institute for Policy Rapid Research Report. 2020 [updated 2020 Jun 10]. Available from: https://www.ipr.northwestern.edu/documents/reports/ipr-rapid-research-reports-pulse-hh-data-10-june-2020.pdf. Accessed 11 Feb 2021.

[66] Rural Health Information Hub. Am I rural? 2020. Available from: https://www.ruralhealthinfo.org/am-i-rural. Accessed 11 Jan 2020.

[67] Emerson RM, Fretz RI, Shaw LL. Writing ethnographic fieldnotes. Chicago: University of Chicago Press; 2011 Dec 25.

[68] Elo S, Kyngäs H (2008). The qualitative content analysis process. J Adv Nurs..

[69] Saldaña J. The coding manual for qualitative researchers. Los Angeles: Sage; 2015 Nov 2.

[70] Wright J, Evans J, Davies B, Wright J (2004). Post-structural methodologies: the body, schooling and health. Body knowledge and control.

[71] Attride-Stirling J (2001). Thematic networks: an analytic tool for qualitative research. Qual Res..

[72] Creswell JW, Poth CN. Qualitative inquiry and research design: choosing among five approaches. Los Angeles; Sage publications; 2016 Dec 19.

[73] Kaczynski D, Salmona M, Smith T (2014). Qualitative research in finance. Aust J Manag..

[74] Hagmann D, Siegrist M, Hartmann C (2018). Taxes, labels, or nudges? Public acceptance of various interventions designed to reduce sugar intake. Food Policy..

[75] Ortega-Avila AG, Papadaki A, Jago R (2018). Exploring perceptions of the Mexican sugar-sweetened beverage tax among adolescents in north-West Mexico: a qualitative study. Public Health Nutr..

[76] Krukowski CN, Conley KM, Sterling M, Rainville AJ (2016). A qualitative study of adolescent views of sugar-sweetened beverage taxes, Michigan. Prev Chronic Dis.

[77] Pommerehne WW, Schneider F (1978). Fiscal illusion, political institutions, and local public spending. Kyklos..

[78] Park S, McGuire LC, Galuska DA (2015). Regional differences in sugar-sweetened beverage intake among US adults. J Acad Nutr Diet.

[79] Nikpartow N, Danyliw AD, Whiting SJ, Lim HJ, Vatanparast H (2012). Beverage consumption patterns of Canadian adults aged 19 to 65 years. Public Health Nutr..

[80] Julia C, Méjean C, Vicari F, Péneau S, Hercberg S (2015). Public perception and characteristics related to acceptance of the sugar-sweetened beverage taxation launched in France in 2012. Public Health Nutr..

[81] Bombak AE, Colotti T, Riediger ND, Raji D, Eckhart N. Fizzy foibles: examining attitudes towards sugar-sweetened beverages in Michigan. Crit Public Health 2021;31(1):https://doi.org/10.1080/09581596.2019.1680804, 31, 1, 124

[82] Schneider A, Ingram H (1993). Social construction of target populations: implications for politics and policy. Am Political Sci Rev..

[83] Farrell LC, Warin MJ, Moore VM, Street JM (2016). Socio-economic divergence in public opinions about preventive obesity regulations: is the purpose to ‘make some things cheaper, more affordable’ or to ‘help them get over their own ignorance’?. Soc Sci Med..

[84] Schoenberg NE, Howell BM, Swanson M, Grosh C, Bardach S (2013). Perspectives on healthy eating among Appalachian residents. J Rural Health..

[85] Beagan BL, Chapman GE, Johnston J, McPhail D, Power EM, Vallianatos H. Acquired tastes: why families eat the way they do. Vancouver: UBC Press; 2014 Nov 15.

[86] McPhail D, Chapman GE, Beagan BL (2013). The rural and the rotund? A critical interpretation of food deserts and rural adolescent obesity in the Canadian context. Health Place..

[87] Trivedi T, Liu J, Probst JC, Merchant A, Jones S, Martin AB (2015). Obesity and obesity-related behaviors among rural and urban adults in the USA. Rural Remote Health.

[88] Warin M (2018). Information is not knowledge: cooking and eating as skilled practice in Australian obesity education. Aus J Anthropol..

[89] Jilcott Pitts SB, Smith TW, Thayer LM, Drobka S, Miller C, Keyserling TC, Ammerman AS (2013). Addressing rural health disparities through policy change in the stroke belt. J Public Health Manag Pract..

[90] Chaloupka FJ, Yurekli A, Fong GT (2012). Tobacco taxes as a tobacco control strategy. Tob Control..

[91] American Lung Association. Tobacco cessation & prevention*.* Chicago, IL; 2017 Jul. Available from: https://www.lung.org/our-initiatives/tobacco/cessation-and-prevention/. Accessed 15 Feb 2019

[92] Hoek J, Smith K (2016). A qualitative analysis of low income smokers’ responses to tobacco excise tax increases. Int J Drug Policy..

[93] Bettigole C, Farley TA (2016). The Philadelphia story: attacking behavioral and social determinants of health. Ann Intern Med..

[94] Langellier BA, Lê-Scherban F, Purtle J (2017). Funding quality pre-kindergarten slots with Philadelphia’s new ‘sugary drink tax’: simulating effects of using an excise tax to address a social determinant of health. Public Health Nutr..

[95] Arango M (2018). A made-in-Canada sugary drink levy can help reduce intake while being mindful of addressing health disparities. CMAJ..

[96] Food Secure Canada. From patchwork to policy coherence: principles and priorities of Canada’s national food policy. Food Secure Canada. 2017 May. Available from: https://foodsecurecanada.org/sites/foodsecurecanada.org/files/201705-from-patchwork-to-policy-coherence-food_secure_canada-discussion-paper-v1.pdf. Accessed 11 Jan 2020.

[97] Backholer K, Sarink D, Beauchamp A, Keating C, Loh V, Ball K, Martin J, Peeters A (2016). The impact of a tax on sugar-sweetened beverages according to socio-economic position: a systematic review of the evidence. Public Health Nutr..

[98] Olstad DL, Ancilotto R, Teychenne M, Minaker LM, Taber DR, Raine KD, Nykiforuk CI, Ball K (2017). Can targeted policies reduce obesity and improve obesity-related behaviours in socioeconomically disadvantaged populations? A systematic review. Obes Rev..

[99] Byrd-Bredbenner C, Wu F, Spaccarotella K, Quick V, Martin-Biggers J, Zhang Y (2017). Systematic review of control groups in nutrition education intervention research. Int J Behav Nutr Phy..

[100] Riediger ND, Bombak AE, Mudryj A, Bensley J, Ankomah S (2018). A systematic search and qualitiative review of reporting bias of lifestyle interventions in randomized control trials of diabetes prevention and management. Nutr J..

[101] Kirkpatrick SI, Raffoul A, Maynard M, Lee KM, Stapleton J (2018). Gaps in the evidence on population interventions to reduce consumption of sugars: a review of reviews. Nutrients..

[102] Leahy D, Wright J, Harwood V (2009). Disgusting pedagogies. Biopolitics and the Obesity Epidemic.

[103] Evans J, Rich E, Davies B, Allwood R (2008). Education, disordered eating and obesity discourse: fat fabrications.

[104] McVey G, Gusella J, Tweed S, Ferrari M (2008). A controlled evaluation of web-based training for teachers and public health practitioners on the prevention of eating disorders. J Eat Disord..

[105] Cawley J, Willage B, Frisvold D (2018). Pass-through of a tax on sugar-sweetened beverages at the Philadelphia International Airport. JAMA..

[106] Silver LD, Ng SW, Ryan-Ibarra S, Taillie LS, Induni M, Miles DR, et al. Changes in prices, sales, consumer spending, and beverage consumption one year after a tax on sugar-sweetened beverages in Berkeley, California, US: a before-and-after study. PLoS Med. 2017;14(4):e1002283. 10.1371/journal.pmed.1002283. 10.1371/journal.pmed.1002283 PMC5395172 28419108

